# Positive effects of the progestin desogestrel 75 μg on migraine frequency and use of acute medication are sustained over a treatment period of 180 days

**DOI:** 10.1186/s10194-015-0522-8

**Published:** 2015-05-01

**Authors:** Gabriele S Merki-Feld, Bruno Imthurn, Ronald Langner, Burkhardt Seifert, Andreas R Gantenbein

**Affiliations:** Department of Reproductive Endocrinology, University Hospital Zürich, Rämistrasse 100, CH - 8091 Zürich, Switzerland; Headache Clinic Hirslanden Zürich, Zurich, Switzerland; Epidemiology, Biostatistics and Prevention Institute, Department of Biostatistics, University of Zürich, Zurich, Switzerland; Neurorehabilitation, RehaClinic, Bad Zurzach, Switzerland

**Keywords:** Hormonal migraine, Contraception, Progestagen-only pill, Desogestrel, Migraine without aura, Headache, Migraine with aura, Cardiovascular risk, Triptans

## Abstract

**Background:**

Premenopausal migraines frequently are associated with fluctuations of estrogen levels. Both, migraine and combined hormonal contraceptives (CHC) increase the risk of vascular events. Therefore progestagen-only contraceptives (POC) are a safer alternative. A previous short-term study demonstrated a positive impact of the oral POC desogestrel on migraine frequency. To study the effect of the POC desogestrel 75 μg on migraine frequency, intensity, use of acute medication and quality of life in a clinical setting over the period of 180 days.

**Methods:**

Patients’ charts were screened for women with migraine, who had decided to use desogestrel for contraception. Charts were included, if routinely conducted headache diaries were complete for 90 days before treatment (baseline) and over a treatment period of 180 days. We also report about starters who stopped treatment early, because of adverse events. Baseline data (day 1–90 before treatment) were compared with first and second treatment period (treatment days 1–90 and days 91–180). Quality of life was evaluated using MIDAS questionnaires.

**Results:**

Days with migraine (5.8 vs 3.6), with any kind of headache (9.4 vs 6.6), headache intensity (15.7 vs 10.7), days with severe headache (5.4 vs 2.4) and use of triptans (12.3 vs7.8) were significantly reduced after 180 days. MIDAS score and grade improved significantly.

**Conclusion:**

Contraception with desogestrel 75 μg resulted in a significantly improved quality of life and a reduction of migraine days over the observation period of 180 days. A clinically meaningful 30% reduction in pain was observed in 25/42 (60%) participants. For counselling reasons it is of importance, that the major reduction in migraine frequency occured during the initial 90 days, however further improvement occurs with longer duration of use. Prospective studies are needed to confirm these results.

## Background

Epidemiological data suggest that combined hormonal contraceptives (CHC) initiate or worsen migraine and headache in predisposed women [1-5]. The incidence of migraine is highest during the reproductive years and more than 50% of women report an association between migraine attacks and their menstrual cycle [6,7]. The reproductive phase is also the life span in which most women need efficient contraception. Migraine with aura (MA) and to a lesser extent migraine without aura (MO) increase the risk for cardiovascular events, especially for stroke [8-11]. There is a substantial elevation of these risks in migraineurs using CHC [11-14]. The cardiovascular risk associated with CHC, has been mainly attributed to the estrogen component which exerts a strong effect on the coagulation system. Finding a well-tolerated estrogen-free form of contraception for headache patients therefore is an important issue.

Progestagen-only pills (POP) have so far not been found to be associated with an increased risk for thromboembolic or ischemic events [15]. Most guidelines recommend progestagen-only contraception as a safer option [16]. The POP desogestrel 75 μg (Cerazette®; MSD Merck Sharp & Dohme AG, Luzern, Switzerland) is used continuously and combines efficient inhibition of ovulation with maintenance of low estrogen levels [17,18]. Avoidance of estrogen peaks and withdrawal could contribute to good tolerability of this contraceptive in migraineurs. Recently we reported a benefit of desogestrel 75 μg on migraine and quality of life over a 3 month period of use [19,20]. The effect on frequency and quality of life was comparable to improvements observed with prophylactic agents. However, the observation interval was short. In the present study, we report effects of 6 cycles desogestrel contraception on headache frequency, intensity and use of pain medication.

## Methods

This study was performed at the divison for family planning, unit of the Department of Reproductive Endocrinology, University Hospital Zürich, Switzerland where one of the authors (GM) runs an outpatient clinic for migraine patients with need for hormonal therapy. Migraine is diagnosed according to the IHS (International Headache Society) criteria by the referring neurologists from headache centres in Zürich, Bad Zurzach or by the author [21]. Reasons for referral were need for contraception in women with migraine, menstrual migraine or any form of hormonal therapy of headaches. To allow an exact diagnosis of the headache type and frequency according to the IHS our patients are principally instructed to conduct headache diaries for 3 cycles before their first visit and to continue after any intervention. MIDAS questionnaires are used before interventions and in intervals of 90 days thereafter. The majority of our premenopausal patients have a need for efficient contraception. In the context of the discussions around the elevated risks for cardiovascular disease and stroke we advise against combined hormonal contraceptives as a first choice contraception in migraineurs and in women aged 35 years or more. Before starting a hormonal treatment women are informed about risks and potential side effects which include information about irregular bleeding and acne with the use of desogestrel.

For the present study patients’ charts were screened for women with migraine, who had decided to use the POP desogestrel 75 μg and had conducted headache diaries 90 days before initiation and over 180 days of use of this medication. We included patients suffering from all types of migraine. The observation period was defined from July 2009 to December 2013. In a previous study we already reported 90 day treatment data of 16 included patients. Women had to be premenopausal and had to need effective contraception. We report about all adverse events causing discontinuation earlier than 180 days. Exclusion criteria were: incomplete diaries, less than 10 headache episodes during the pretreatment period, initiation or change of prophylactic medications during the observation and postmenopause. This resulted in a drop-out rate of 26 out of 68 charts.

The diaries include information on the number of migraine and headache days, the severity of headache, the use of triptans and other pain medication, the use of hormones and days with vaginal bleeding. Days of bleeding were assessed to allow an exact diagnosis of the migraine type according to the IHS criteria. Headache severity was rated in the diaries according to a 4-point scale (0 = no pain, 3 = severe pain). This score is easy to understand and has been proven to be useful in daily work with migraineurs. For ethical reasons all diaries were anonymised before data evaluation. The evaluation of anonymised data in our setting was accepted by the ethical committee of the Kanton Zürich.

Primary efficacy variables were the differences in number of migraine and headache days, the difference in pain score as well as MIDAS score and grade. Secondary outcomes included differences in the number of all pain medications and triptans used as well as differences in days with pain score three. In population-based studies of migraine- and headache sufferers in the US and UK the MIDAS questionnaire and the MIDAS summary scores proved to be a highly reliable means of assessment of the impact of the ailment on daily life [22,23]. The total MIDAS score strongly correlates with both the clinical evaluation of the severity of a patient’s headache problem, and the frequency of the episodes, determined from daily-based headache diaries [23].

### Statistical analyses

Data were compared between baseline (BL) (day 1–90 before treatment) and treatment periods (TP): TP1 (day 1–90) TP2 (day 91–180). In addition all variables were compared between TP1 and TP2. Statistical analyses were done using IBM SPSS Statistics, version 22 (Armonk, New York, IBM Corp). Data are presented as mean (SD). Pain intensity score was calculated as the sum of headache intensities for baseline and each treatment period according to the above mentioned 4-point scale. For each period this sum was divided by three to obtain a mean monthly pain score. To calculate monthly frequencies, the numbers for each observational episode was divided by three. Numbers of monthly migraine days, headache days, headache intensity, days with use of pain medication and questions of the MIDAS questionnaire were compared with Friedman’s test. Post-hoc comparisons between single time points were performed using Wilcoxon’s signed rank test with Bonferroni correction.

## Results

**Figure 1:**
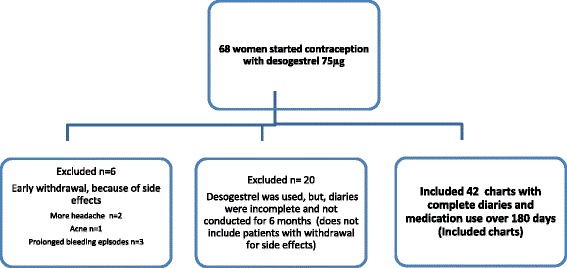
Flow diagram of the study population.

A total of 68 women with migraine initiated contraception with desogestrel 75 μg. Headache diaries of 42 subjects were complete and eligible for analysis. Six patients had stopped desogestrel because of side effects within 42 or less days and were excluded (prolonged bleeding n = 3, increase of headache n = 2, acne = 1) (Figure 1). Demographics and characteristics of eligible women and drop-outs did not differ significantly (Table 1). Hormonal contraception was used by 50% (n = 21) of the included patients and 61% (n = 16) in the drop-out (p > 0.05). One included woman had used a copper-device (drop-outs: n = 0). Chronic headaches (>15 /month) were found in 6 included patients and more than 8 triptans were used monthly by 9 included patients. Mean age of migraine onset was 22.4 years (SD 5.2). Two women suffered from endometriosis. Frequency of migraine, headache intensity, days with use of pain medication and triptans were significantly reduced during TP2 in comparison with BL (Table 2). Days with severe pain declined from 5.4 (SD 4.2) to 2.4 (SD 3.5) (p < 0.001) (Table 2). The improvements were in large parts visible during TP1 and persisted during further follow-up. A according to the IHS clinically meaningful 30% reduction in pain was observed in 25/42 (60%) participants, whereas another 28% (12/42) experienced even a 50% reduction [24,25]. We found a 255 reduction in the sum of headache and migraine days in 55% (23/42) of the included migraineurs. Seven of 42 patients (16%) experienced 1–5 more headache/migraine days during TP2 in comparison to BL. Interestingly, however, quality of life improved in five of these seven women. Further analyses to explain this seemingly contradictory result revealed a decrease in days with pain score 3 and a decrease in overall pain intensity in all these five patients. Two women with more migraine attacks and without improvement in the MIDAS score, decided to change to a non-hormonal contraception after 180 days.

**Table 1:** **Demographic and baseline characteristics of included charts (n = 42) and excluded charts (n = 26)**

**Demographics**	**Included patients n = 42**	**Drop-out group n = 26**	**P - value**
	**Mean (SD)**	**Mean (SD)**	
Age (years)	35.1 (8.9)	31.1 (9.8)	0.64
Height (cm)	165.7 (6.5)	166.2 (5.7)	0.78
Weight (kg)	60.6 (8.4)	61.6 (9.0)	0.37
Systolic blood pressure (mmHG)	119.9 (12.6)	114.7 (26.1)	0.19
Diastolic blood pressure (mmHG)	73.7 (9.9)	75.7 (9.6)	0.91
**Baseline characteristics**			
Migraine days per month	5.8 (4.3)	6.6 (3.6)	0.62
Headache Intensity /month	15.7 (7.6)	20.3 (9.5)	0.28
Age of migraine onset	22.4 (5.2)	24.2 (7.2)	0.42
Triptan users	12.3 (15.0)	12.1 (11.7)	0.71
MIDAS: Headache days	25.2 (17.2)	23.0 (12.8)	0.37
MIDAS: (Pain intensity)	6.4 (1.7)	6.7 (1.6)	0.57
MIDAS: Grade	3.5 (0.7)	3.3 (0.8)	0.38
	Number (%)	Number (%)	
Migraine with aura	10 (24)	10 (38)	0.19
Migraine without aura	32 (76)	16 (61.1)

**Table 2:** **Changes in migraine, frequency, intensity and use of pain medication during use of the contraceptive pill desogestrel 75 μg over 180 days of use**

**Days/month**	**Mean baseline (SD)**	**Mean treatment TP 1(SD)**	**Mean treatment TP 2 (SD)**	**Overall P-value**	**P-value baseline vs. TP 1**	Posthoc* P-value baseline vs. TP2	**P-value TP 1 vs TP 2**
Headache days	4.1 (4.5)	3.8 (4.1)	3.0 (4.1)	0.051	0.867	0.050	0.062
Migraine days	5.8 (4.3)	3.7 (3.4)	3.6 (4.0)	<0.001	<0.001	0.001	0.701
Sum headache and migraine	9.4 (5.1)	7.6 (5.5)	6.6 (5.4)	0.002	0.008	<0.001	0.130
Headache intensity	15.7 (7.6)	11.4 (7.5)	10.7 (8.0)	<0.001	<0.001	<0.001	0.312
Days with headache score 3 in 3 months	5.4 (4.2)	2.2 (2.7)	2.4 (3.5)	<0.001	<0.001	<0.001	0.776
Pain medication	7.2 (5.9)	5.0 (3.3)	5.0 (4.0)	0.044	<0.001	0.015	0.751
Triptan use in 3 months	12.3 (15.0)	8.1 (9.4)	7.8 (10.2)	0.035	0.010	0.041	0.599

Baseline: 90 days before treatment; TP = treatment period. TP1: Treatment period days 1–90; TP2: Treatment period days 91–180.

*after Bonferroni correction, post-hoc p-values are significant at p < 0.017.

Table 3 demonstrates the changes in quality of life. All MIDAS items improved significantly during 180 days of desogestrel use (TP2). Again significant improvement was already observed after TP1. Separate analyses for MO and MA women revealed no differences with regard to demographic parameters between the groups. In MO patients significant improvements of all features (except headache days) days were observed (Table 4). The very small group of subjects with MA experienced significant reductions in the number of pain medications and triptans, MIDAS score and MIDAS grade.

**Table 3:** **Changes in quality of life measured with the MIDAS after 90 days and 180 days contraception with desogestrel 75 μg**

**N = 42**	**Mean (SD) Baseline**	**Mean (SD) TP 1**	**Mean (SD) TP 2**	**Overall p-value**	**P-value baseline vs. TP1**	Posthoc* P-value baseline vs. TP2	**P-value TP1 vs. TP2**
MIDAS SCORE	36.3 (41.9)	18.3 (38.8)	16.0 (32.8)	<0.001	<0.001	<0.001	0.176
MIDAS 1: days missed at work	7.0 (15.2)	3.8 (13.6)	2.6 (10.9)	<0.001	<0.001	0.002	0.093
MIDAS 2: days with >50% reduced productivity at work	7.6 (5.4)	4.2 (4.6)	3.9 (4.9)	0.001	0.002	0.001	0.650
MIDAS 3: days without household work	6.3 (9.7)	3.6 (8.1)	2.6 (4.4)	<0.001	0.002	<0.001	0.161
MIDAS 4: days with >50% reduced productivity in household work	5.8 (6.8)	3.9 (7.0)	3.9 (8.2)	0.006	<0.009	0.046	0.766
MIDAS 5: days when family, social or leisure activities are missed	9.8 (15.3)	3.7 (9.9)	3.1 (7.2)	<0.001	<0.001	<0.001	0.481
MIDAS: Headache days	26.4 (19.3)	17.0 (15.5)	17.0 (18.1)	<0.001	0.002	<0.001	0.487
MIDAS: Pain intensity (scale 0–10)	6.1 (1.7)	4.8 (1.5)	4.5 (2.0)	<0.001	<0.001	<0.001	0.078
MIDAS: Grade	3.6 (0.7)	2.4 (1.0)	2.2 (1.2)	<0.001	<0.001	<0.001	0.295

Baseline: before treatment, TP1: 1–90 days use of desogestrel; TP2: 91–180 days desogestrel use.

*after Bonferroni correction, post-hoc p-values are significant at p < 0.017.

**Table 4:** **Changes in migraine and headache frequency during use of the progestin-only pill desogestrel 75 μg, comparison between MO (n=32) and MA (10) patients**

	**MO Mean (SD) baseline**	**MA Mean (SD) baseline**	**MO Mean (SD) TP 2**	**MA Mean (SD) TP 2**	**p-value MO baseline vs. TP 2**	**p-value MA baseline vs. TP 2**
Headache days/month	4.1 (4.3)	3.8 (5.2)	3.4 (4.6)	1.9 (2.0)	0.06	0.60
Migraine days/month	5.4 (4.1)	6.8 (4.8)	3.2 (3.8)	4.6 (4.3)	0.007	0.09
Sum headache and migraine/month	9.5 (5.6)	8.8 (3.6)	6.6 (4.4)	6.6 (4.4)	<0.001	0.11
Headache intensity/month	16.0 (8.3)	14.4 (5.4)	11.4(8.4)	8.5 (6.4)	<0.001	0.24
Pain medication	6.2 (3.6)	9.8 (9.5)	4.7 (3.6)	5.8 (4.8)	0.03	0.03
Triptan use in 3 months	11.9 (11.8)	13.1 (22.2)	8.4 (10.7)	6.2 (8.6)	0.005	0.02

MO = migraine without aura, MA = Migraine with aura, baseline : days 1–90 before treatment; TP 2: days 91–180 of treatment.

## Discussion

In the present study we report the effects of 180 days of contraception with the progestin-only pill desogestrel 75 μg on headache and migraine. We observed a significant reduction in migraine frequency, migraine intensity, use of triptans and pain score. Quality of life measured by the MIDAS score improved by more than 50%. Mean MIDAS grades were diminished by point (Table 3). The majority of positive effects were apparent after 90 days and small further improvements were noted up to 180 days of use (Table 2). To our knowledge, we report for the first time that hormonal treatment can reduce the use of triptans significantly. This might be of relevance for women at the boarder of medication overuse headaches. As different pathophysiologies underlie MA and MO, we performed subanalyses for both types of headaches. In women with MO, significant improvements for all variables except headache days were observed. In the group of patients suffering from MA (n = 10) migraine days decreased by two/month, what possibly as a result of the small group size was not significant. Significant bettermends were observed with regard to use of pain medications, use of triptans and MIDAS score and grade.

Migraine is a typical disorder with a high response rate to placebo in controlled trials. For ethical reasons placebo-controlled studies in the area of contraception are not acceptable. An important strength of the present study is the long run-in period and the evaluation not only of migraine frequency but also of additional parameters, like pain intensity, use of pain medications and quality of life. The combination of these data is a better reflection of the overall well-being as demonstrated in the detailed data analysis of the patients developing more migraine in our study. The run in period of 90 days allowed a balanced overview with regard to migraine frequencies which can vary markedly from month to month. However, even if the headache diaries had been conducted prospectively our analyses could have generated selection or information bias. In particular, we assume that a prospective design might have resulted in a higher continuation rate and exclusion of less charts with incomplete diaries. A control group of women using other hormonal contraceptive methods would have been of advantage.

Our findings for MO patients are in accordance with a very recent retrospective diary-based study, demonstrating a significant reduction in migraine frequency, pain intensity and use of pain medication with 6 months use of desogestrel [26]. Triptan use did not decline, which contrasts with our result and might be related to the lower number of included patients. The comparison with a control group of users of a combined pill (COC) in a long-cycle in this study is of great interest, because both forms of contraception prevent hormone withdrawal [26]. While migraine attacks and pain intensity decreased significantly with the POP, headache frequency declined with the COC regimen only. Desogestrel use failed to exert a significant effect on non-migrainous-headache in our sample and the comparative trial. This can be explained, by our earlier reported findings showing in an individual follow-up of both headache and migraine, that a temporary transformation of migraines to headaches occurs in some women [19]. Our present study with a longer observation period however indicates that, on the long-term, these headaches might decline as well (p = 0.05). Although our study includes only few women with MA, the findings are backed by Nappi et al. who reported a significant reduction in migraine frequency in MA patients, but did not investigate pain intensity and quality of life [27].

Even if there is still a lack of prospective controlled trials several diary-based studies indicate a positive impact of desogestrel on migraine without aura [19,26,27]. Continuous use of COCs exerts a positive impact on headaches and hormone-withdrawal migraines, however POP are much safer with regard to the cardiovascular and thromboembolic risks [26,28-32]. The benefit of desogestrel on migraine with aura, which is not typically associated with estrogen withdrawal, has to be confirmed in future studies. Many migraineurs are reluctant to use hormones as a consequence of previous bad experience. During counselling it is helpful to know that major improvements can be expected during 90 days of desogestrel use. Furthermore, two trials indicate that migraines and pain tend to improve further beyond 3 months [19,26,27]. On the other hand, patients have to be informed that migraine rarely worsens. The clinically meaningful 30% reduction in pain (considered by the IHS as clinically meaningful) in 60% of our patients is another argument to prefer this contraceptive method in women with migraine [24,25]. Reduction in use of triptans and other pain medications might contribute of the prevention of medication-overuse headaches.

In daily life the degree to which a reduction in headache frequency translates to decreased disability and improved quality of life is highly relevant. The MIDAS demonstrated highly reduced disability and significantly improved quality of life in our patients.

Use of POP can cause a variety of bleeding patterns including amenorrhoea, infrequent bleeding, frequent bleeding and prolonged bleeding episodes. Unfavorable bleeding patterns such as frequent bleeding and prolonged bleeding occur as result of the continuous progestin effect on the endometrium and can be a reason for withdrawal from this form of contraception [33]. Prolonged and frequent bleedings usually stop with longer duration of use and can be treated if not.

### Unanswered questions

New insights in the hormonal effects on the brain allow speculations about mechanisms underlying our observations. Avoidance of hormone withdrawal can only explain the decline of cycle-related headaches. In contrast to estrogens, progesterone seems to attenuate trigeminovascular nociception and reduces dural plasma protein extravasation following stimulation of the trigeminal ganglion [34-36]. Thus direct or receptor-mediated effects of the desogestrel on the trigeminovascular system can be postulated. The variety of responses on desogestrel treatment could be a result of the genetic variability of estrogen receptors in women, with some polymorphisms being a significant risk factor for migraine [37]. The neurological basis of migraine auras has not yet been established, increasing evidence indicates that they are a clinical manifestation of a cortical spreading depression (CSD).

In mice the thresholds for cortical spreading depression (CSD) is lower in cycling females than in males. This would allow to hypothesise that maintenance of low estrogen levels induced by desogestrel might upregulate the threshold for CSD thus reduce MA attacks. A further mechanism could be that desogestrel or its metabolite etonogestrel, like progesterone and allopregnanolone decrease cortical excitability via the GABA -receptor [38-40].

At the moment we have no means to predict how an individual migraineur will react on desogestrel. Outside the study we achieved positive effects with higher dosages. However, this is off-label use and cannot be generally recommended before prospective trials have been conducted. Several trials highlight a positive effect of desogestrel on migraine. Among neurologists it is well known that headaches may be cycle-related, but they rarely consider to search advice for a hormonal treatment. Vice versa gynaecologists are not always aware of the fact that hormonal treatment affects headache frequency in predisposed women. A closer collaboration between gynaecologic endocrinologists and headache specialists might provide better care and safety for young women, suffering from migraine during use of any hormones or in association with their natural cycle.

## Conclusion

In conclusion our data indicate a positive impact of desogestrel 75 μg on migraine frequency, intensity, use of pain medication and quality of life. The major improvement was observed during the initial 90 days of use, which might be important for patients’ counselling. Randomised controlled trials are needed to substantiate our results.
